# Intralesional injection of rose bengal augments the efficacy of gemcitabine chemotherapy against pancreatic tumors

**DOI:** 10.1186/s12885-021-08522-z

**Published:** 2021-06-30

**Authors:** Patrick Innamarato, Jennifer Morse, Amy Mackay, Sarah Asby, Matthew Beatty, Jamie Blauvelt, Scott Kidd, John E. Mullinax, Amod A. Sarnaik, Shari Pilon-Thomas

**Affiliations:** 1 Department of Immunology, H. Lee Moffitt Cancer Center and Research Institute, 12902 USF Magnolia Dr, Tampa, FL 33612 USA; 2 Cancer Biology Ph.D. Program, University of South Florida, Tampa, FL USA; 3 Sarcoma Department, H. Lee Moffitt Cancer Center, 12902 Magnolia Drive, Tampa, FL 33606 USA; 4 Department of Cutaneous Oncology, H. Lee Moffitt Cancer Center, Tampa, FL USA

## Abstract

**Background:**

Chemotherapy regimens that include the utilization of gemcitabine are the standard of care in pancreatic cancer patients. However, most patients with advanced pancreatic cancer die within the first 2 years after diagnosis, even when treated with standard of care chemotherapy. This study aims to explore combination therapies that could boost the efficacy of standard of care regimens in pancreatic cancer patients.

**Methods:**

In this study, we used PV-10, a 10% solution of rose bengal, to induce the death of human pancreatic tumor cells in vitro. Murine in vivo studies were carried out to examine the effectiveness of the direct injection of PV-10 into syngeneic pancreatic tumors in causing lesion-specific ablation. Intralesional PV-10 treatment was combined with systemic gemcitabine treatment in tumor-bearing mice to investigate the control of growth among treated tumors and distal uninjected tumors. The involvement of the immune-mediated clearance of tumors was examined in immunogenic tumor models that express ovalbumin (OVA).

**Results:**

In this study, we demonstrate that the injection of PV-10 into mouse pancreatic tumors caused lesion-specific ablation. We show that the combination of intralesional PV-10 with the systemic administration of gemcitabine caused lesion-specific ablation and delayed the growth of distal uninjected tumors. We observed that this treatment strategy was markedly more successful in immunogenic tumors that express the neoantigen OVA, suggesting that the combination therapy enhanced the immune clearance of tumors. Moreover, the regression of tumors in mice that received PV-10 in combination with gemcitabine was associated with the depletion of splenic CD11b^+^Gr-1^+^ cells and increases in damage associated molecular patterns HMGB1, S100A8, and IL-1α.

**Conclusions:**

These results demonstrate that intralesional therapy with PV-10 in combination with gemcitabine can enhance anti-tumor activity against pancreatic tumors and raises the potential for this strategy to be used for the treatment of patients with pancreatic cancer.

## Introduction

The intralesional injection of PV-10 can induce the destruction of injected tumors and simultaneously induce a systemic immune response that promotes the regression of distal, uninjected tumors [1, 2]. PV-10 is a solution of the xanthene dye, rose bengal disodium, that is currently being investigated in multiple clinical trials as an anti-cancer agent for multiple malignancies including cutaneous melanoma (NCT02557321) and metastatic liver cancer (NCT00986661) [3, 4].

Previous reports from our group have demonstrated that the direct injection of PV-10 into murine melanoma tumors can completely eliminate injected lesions and can also promote the regression of distal uninjected (“bystander”) lesions in the skin and lungs [1]. We demonstrated that the release of the damage associated molecular pattern (DAMP), high mobility group box 1 (HMGB1), from PV-10 injected tumors induced the activation of dendritic cells (DCs) which subsequently primed anti-tumor T cell responses in lymph nodes. Moreover, treatment with PV-10 in melanoma patients resulted in increased levels of HMGB1 in the serum, which was associated with improved anti-tumor activity of circulating T cells. Thus, in addition to its direct ablative properties, PV-10 is effective at inducing DAMP release from tumors which can augment anti-tumor immune responses.

The clearance of tumors after treatment with radiotherapy and chemotherapy agents is dependent on the induction of immunogenic cell death and the release of DAMPs [5]. However, the immunologic consequences of DAMP release can have disparate effects on anti-tumor immunity. Indeed, HMGB1 promotes the cross-presentation activity of DCs required for T cell priming [6]. Yet, HMGB1 also promotes the accumulation and immunosuppressive capacity of myeloid derived suppressor cells (MDSCs) [7, 8]. Similarly, the DAMP, heat shock protein 70 (Hsp70) can prevent the translocation of peptide-major histocompatibility complexes (pMHC) and promote the production of interleukin 10 (IL-10), which subsequently suppresses anti-tumor T cell responses [9, 10]. In contrast, Hsp70 also promotes natural killer (NK) cell-mediated cytotoxicity of tumor cells [11]. Thus, the characterization of the DAMP release and its effects on the immune system are necessary to understand therapeutic responses to anti-cancer agents.

Despite the marked improvement in therapeutic options for development of multiple tumor types over the past decade, pancreatic cancer has remained difficult to treat and only 4% of patients live beyond 5 years after their initial diagnosis [12–14]. Furthermore, frontline treatments that include the utilization of gemcitabine have short-term benefits, but ultimately lead to chemoresistance and disease progression [15–18]. The robust therapeutic resistance of pancreatic cancer is due in part to the architecture of the tumor and the frequency of disseminated disease at the time of clinical presentation. For instance, the pancreatic tumor stroma can promote T cell exclusion and also harbors a highly immunosuppressive microenvironment, which induces T cell dysfunction [19–22]. Moreover, tumor-infiltrating T cells are localized to stromal elements of tumors and are spatially distant from pancreatic tumor cells [23]. Thus, it is imperative to develop new therapeutic strategies to augment standard of care practices with a goal of promoting anti-tumor immunity against local and distant pancreatic tumors.

In this study, we investigated the efficacy of intralesional (i.l.) PV-10 treatment in combination with gemcitabine in pancreatic tumor models. We demonstrate that PV-10 can effectively augment the efficacy of gemcitabine against murine pancreatic tumors. This combination strategy may lead to an improved therapy for patients with pancreatic cancer.

## Results

### PV-10 kills pancreatic tumor cells in vitro

**Fig. 1:**
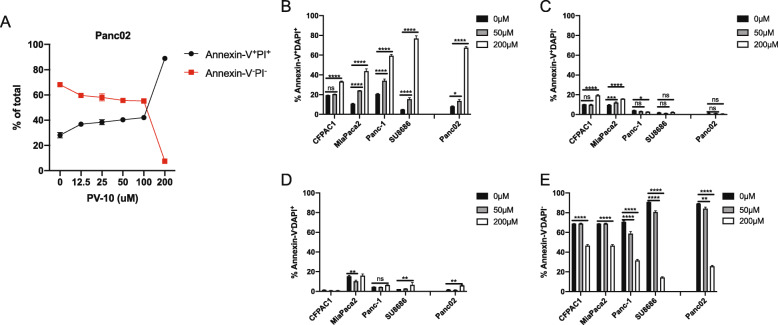
PV-10 kills mouse and human pancreatic tumor cells in vitro. (**A-E**) Mouse Panc02 tumor cells and human pancreatic tumor cell lines, CFPAC-1, MiaPaca2, Panc-1, and SU8686, were cultured overnight with the indicated concentrations of PV-10. (**A**) Percentage of Annexin-V^+^PI^+^ and Annexin-V^−^PI^−^ Panc02 cells. Percentage of (**B**) Annexin-V^+^DAPI^+^, (**C**) Annexin-V^+^DAPI^−^, (**D**) Annexin-V^−^DAPI^+^, (**E**) Annexin-V^−^DAPI^−^ mouse and human pancreatic tumor cells after PV-10 treatment

We first determined whether PV-10 could effectively kill pancreatic tumor cells. We cultured murine Panc02 tumor cells with varying concentrations of PV-10 for 24 h and determined that a concentration of 200 μM was the most effective at inducing cell death as determined by Annexin-V and DAPI positivity (Fig. 1A). We next cultured a panel of human pancreatic tumor cell lines (CFPAC1, MiaPaca2, Panc-1, and SU8686) and murine Panc02 in media without PV-10, with 50 μM PV-10, or with 200 μM PV-10. We found that 200 μM PV-10 was highly effective at inducing cell death as indicated by Annexin-V and DAPI double-positive cells, albeit the frequency of dead cells varied amongst individual tumor cell lines. Similarly, the frequency of live cells (Annexin-V^−^DAPI^−^) were decreased in all tested tumor cell lines after treatment with 200 μM PV-10. Moreover, PV-10 modestly increased the frequency of Annexin-V^+^DAPI^−^ and Annexin-V^−^DAPI^+^ cells in some tumor cell lines. Additionally, the 50 μM PV-10 increased cell death compared to untreated tumor cells, but at a lower rate in comparison to cells treated with 200 μM PV-10 (Fig. 1B-E).

**Fig. 2:**
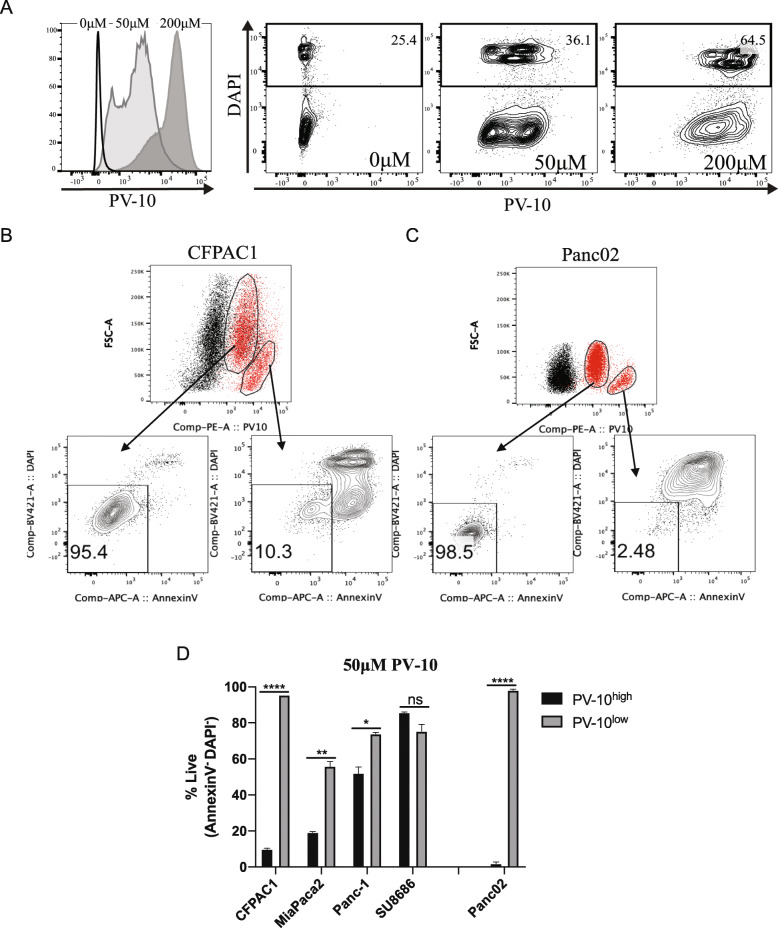
PV-10 saturation is an indicator of cell death (**A**) Staining intensity of PV-10 on Panc-1 cells (histogram on far left) and the frequency of DAPI^+^ dead cells (dot plots to the right of the histogram). (**B-C**) Representative dot plots showing increased cell death in (**B**) CFPAC-1 cells and (**C**) Panc02 cells with high PV-10 uptake (PV-10^high^) and low PV-10 uptake (PV-10^low^). Black population on dot plots are unstained, untreated control cells. Red populations are cells cultured with 50 μM PV-10. Arrows indicate the subgate of each respective population. (**D**) Percentage of live PV-10^high/low^ cells treated with 50 μM PV-10

We next determined that the saturation of PV-10 in tumor cells was associated with increased cell death. Rose bengal excites at 525 nm and emits a fluorescent signal at 570 nm, which allowed us to assess tumor cell uptake of PV-10. Notably, the staining intensity corresponded to the frequency of dead cells. We observed that the frequency of dead Panc-1 cells increased after treatment with 200 μM PV-10 and that the staining intensity was higher in comparison to Panc-1 cells treated with 50 μM PV-10 (Fig. 2A). In cells treated with 50 μM PV-10, we identified two distinct populations of tumor cells that had differing intensities of PV-10 staining and uptake (PV-10^high^ and PV-10^low^). In most tumor cells lines, the frequency of live cells was significantly reduced in PV-10^high^ tumor cells in comparison to PV-10^low^ cells. Moreover, dead cells were almost exclusively contained within the PV-10^high^ population (Fig. 2B-D). Together, these data demonstrate that PV-10 can effectively kill human and murine pancreatic tumor cells.

### In vivo efficacy of intralesional PV-10 against murine pancreatic tumors

**Fig. 3:**
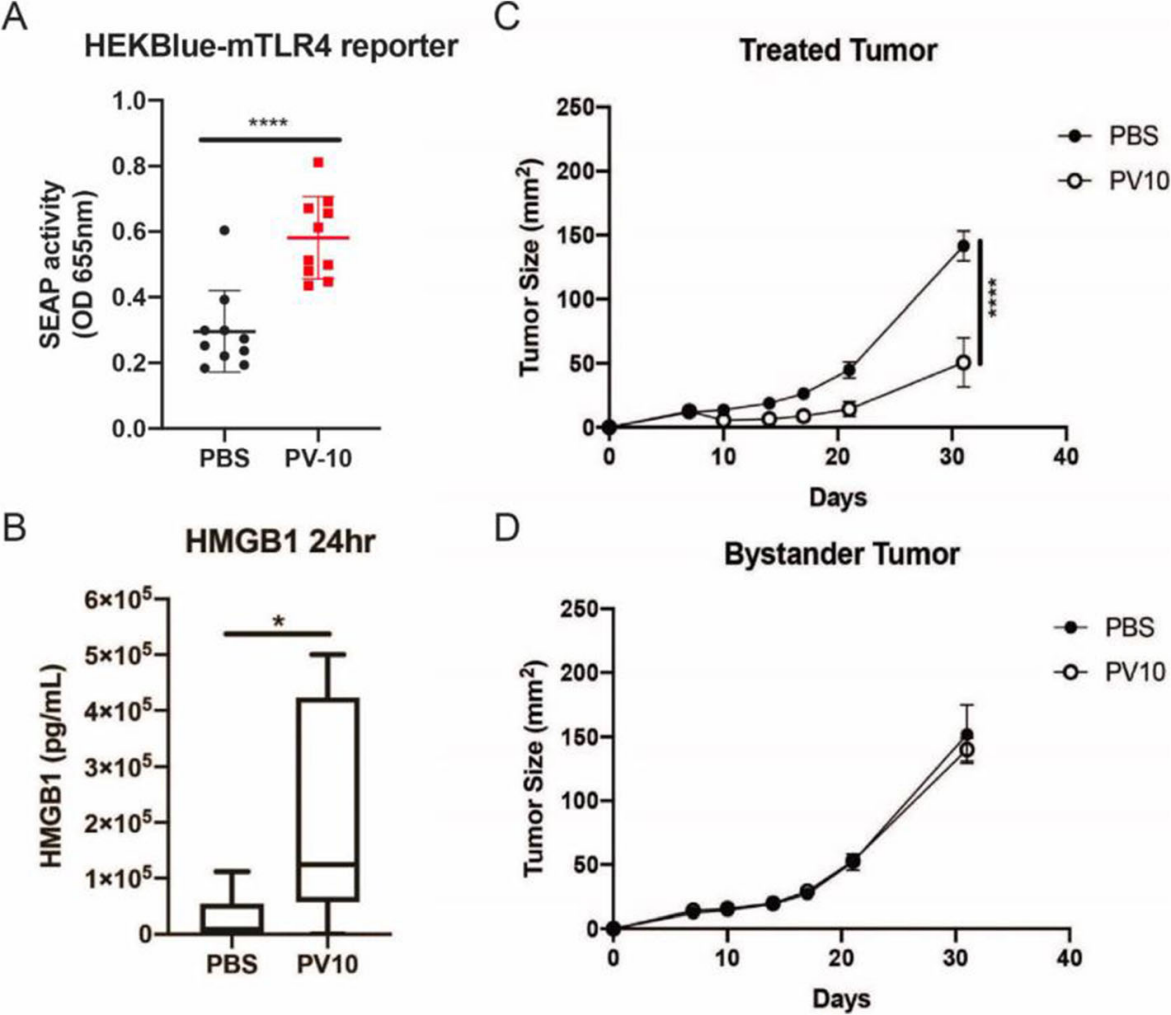
Effect of PV-10 against murine Panc02 tumors in vivo*.* (**A**) Serum from mice treated with i.l. PBS or i.l. PV-10 were collected 24 h after treatment. Serum was cultured with HEKBlue-mTLR4 reporter cells overnight. (**B**) HMGB1 in the sera of mice treated with i.l. PBS or i.l. PV-10. (**C**) Tumor growth of tumors treated with i.l. PBS or i.l. PV-10. (D) Tumor growth of uninjected tumors implanted on the opposite flank of mice

First, we evaluated whether the release of DAMPs could be associated with tumor regressions in response to PV-10 treatment. TLR4 is a receptor for lipopolysaccharide (LPS), but also for the DAMP, HMGB1. We used a reporter cell line to assess the activation of TLR4. Serum was collected from PV-10 treated mice or control PBS treated mice 24 h after intralesional (i.l.) injection and cultured with HEK-Blue mTLR4 cells overnight. TLR4 reporter activity was increased in cells exposed to serum from PV-10 treated mice (Fig. 3A). Likewise, the abundance of HMGB1 was increased in the serum of mice 24 h after PV-10 treatment, suggesting that HMGB1 could activate TLR4 in PV-10 treated mice (Fig. 3B). Next, we assessed the activity of PV-10 in a bilateral model in mice bearing Panc02 tumors. One tumor was injected with i.l. PBS or PV-10, while the tumor on the opposite flank was left untreated. PV-10 treatment slowed tumor growth in injected tumors but had no effect on uninjected bystander tumors (Fig. 3C-D).

### Gemcitabine treatment is enhanced by intralesional PV-10

**Fig. 4:**
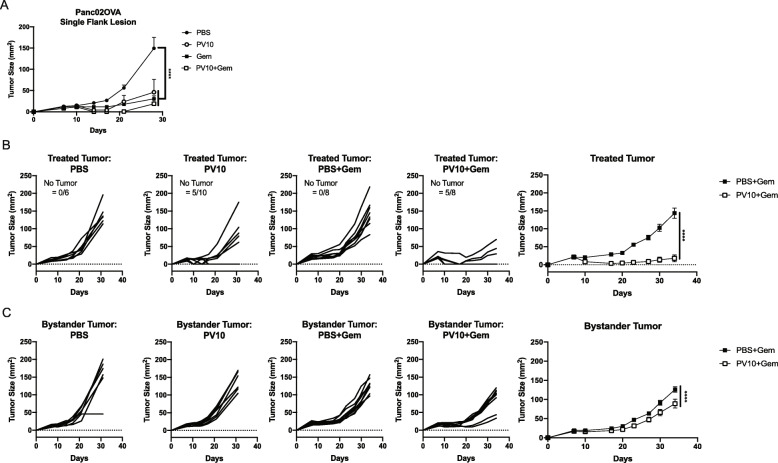
Panc02OVA tumors are responsive to PV-10, gemcitabine combination therapy. (**A**) Tumor growth in mice bearing a single s.c. Panc02OVA tumor treated with i.l. PV-10 ± i.p. gemcitabine (Gem). (**B-C**) Tumor growth curves of individual mice with bilateral Panc02OVA tumors. (**B**) Tumor growth curves for mice treated with i.l. PBS or i.l. PV-10 ± i.p. Gem. Summary of growth among treated tumors with i.l. PBS or i.l. PV-10 in combination with Gem (far right). (**C**) Growth curves for uninjected contralateral tumors among individual mice. Summary of growth among contralateral tumors in mice that received i.l. treatment in the opposite flank in combination with i.p. Gem (far right). Sample size is indicated on each graph in (**B**). The rate of complete regression is also indicated on each graph in (**B**)

PV-10 effectively delayed tumor growth after i.l. injection into Panc02 tumors, but did not promote a systemic immune response that could elicit anti-tumor activity in uninjected tumors. We hypothesized that PV-10 would effectively induce a systemic immune response to pancreatic tumor cells that expressed a highly immunogenic antigen. In mice with a single s.c. Panc02 tumor expressing the ovalbumin (OVA) protein, we found that i.l. treatment with PV-10 was as effective as treatment with systemic gemcitabine or the combination of i.l. PV-10 and gemcitabine (Fig. 4A). However, in a bilateral s.c. tumor model, gemcitabine treatment alone failed to reduce tumor growth in either lesion, suggesting that the increased tumor burden reduced the efficacy of the chemotherapy. In contrast, i.l. PV-10 treatment alone and the combination of i.l. PV-10 with systemic gemcitabine induced complete regression in 50 and 62.5% of treated tumors respectively (Fig. 4B). In uninjected bystander tumors, tumor growth was effectively delayed in mice that received i.l. PV-10 in one lesion in combination with systemic gemcitabine (Fig. 4C). Together, these data suggest that tumor burden and the immunogenicity of pancreatic tumors affect the efficacy of combinatorial i.l. PV-10 and systemic gemcitabine.

**Fig. 5:**
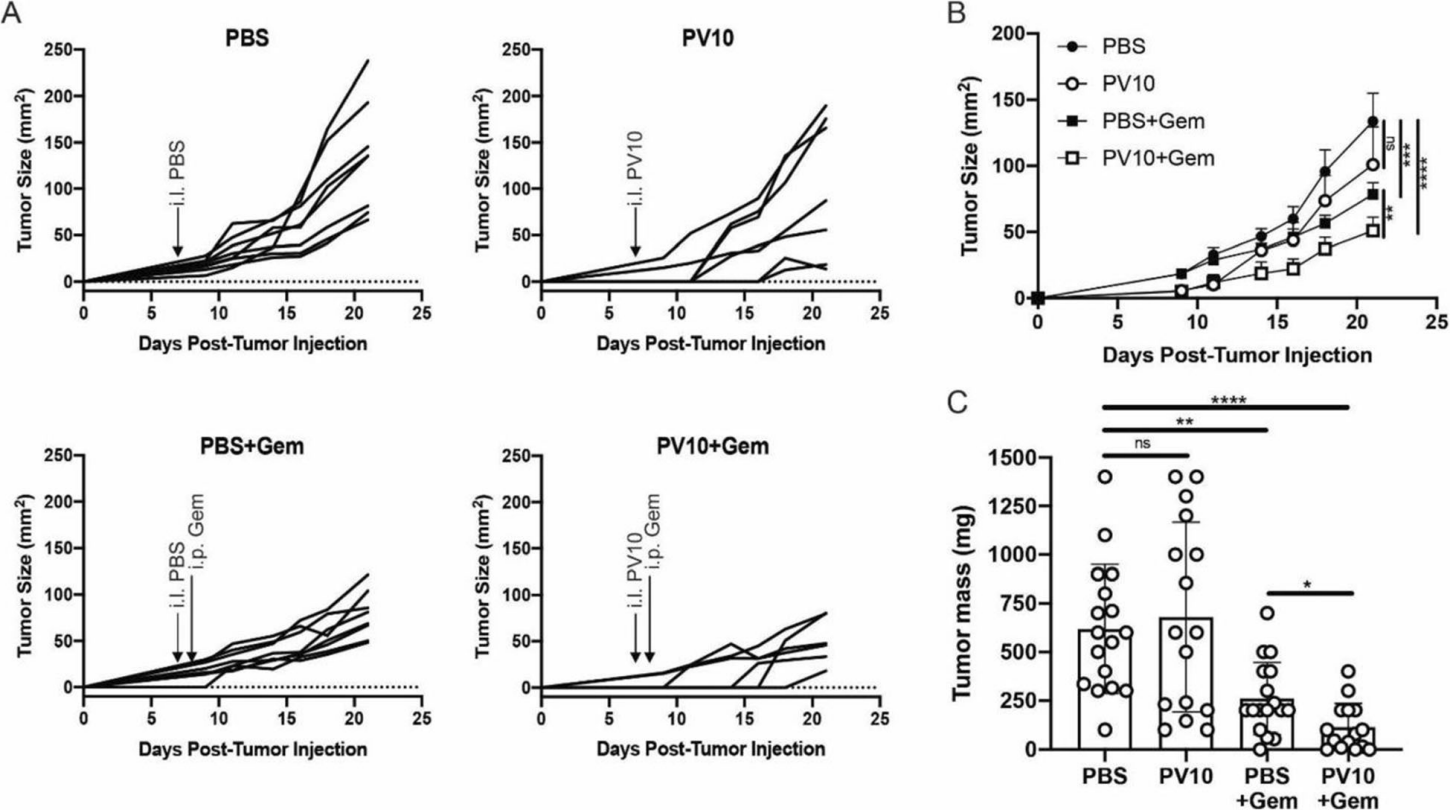
Combination therapy with PV-10 and gemcitabine induces tumor regression. (**A**) Individual tumor growth curves in mice that received i.l. PBS (top left), i.l. PV-10 (top right), i.l. PBS + i.p. Gem (bottom left), i.l. PV-10 + i.p. Gem (bottom right). (**B**) Summary of tumor growth curves from (**A**) (*n* = 6–8 per group). Data are representative of 2 independent experiments (**C**) The mass of tumors at the termination of the experiment. Data are a compilation of 2 independent experiments. (*n* = 16–17 per group)

In a single-flank model in mice with Panc02 tumors, we observed that i.l. PV-10 alone delayed tumor growth in a subset of mice in comparison to mice that received i.l. PBS. Moreover, systemic gemcitabine delayed tumor growth, but the combination of i.l. PV-10 with systemic gemcitabine was the most effective at delaying tumor growth (Fig. 5A-B). Next, we harvested tumors at the termination of the experiment and confirmed that mice treated with i.l. PBS and systemic gemcitabine had smaller tumors in comparison to mice that received i.l. PBS alone or PV-10 alone. Moreover, tumors were significantly smaller in mice that received the combination treatment of PV-10 with systemic gemcitabine (Fig. 5C). Thus, these data demonstrate that i.l. PV-10 enhances the efficacy of systemic gemcitabine treatment.

### Gemcitabine reduces the frequency of peripheral myeloid cells

**Fig. 6:**
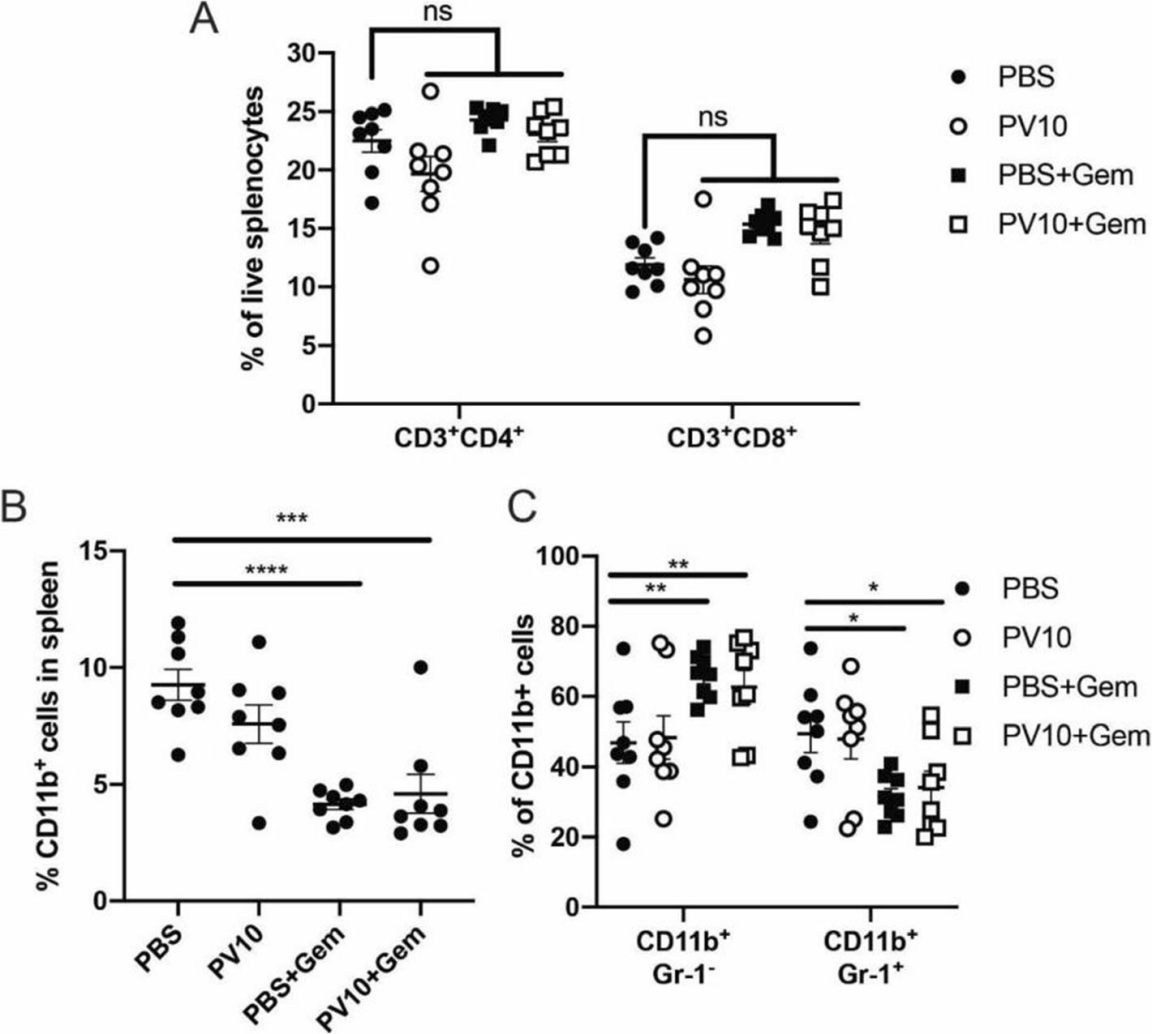
Gemcitabine reduces the frequency of peripheral myeloid cells. (**A**) The frequency of CD3^+^CD4^+^ and CD3^+^CD8^+^ T cells in spleens. (**B**) The frequency of CD11b^+^ myeloid cells in spleens is reduced after Gem treatment. (**C**) The percentage of cell subsets CD11b^+^Gr-1^+/−^ among total myeloid cells in spleens. (*n* = 8 per group)

**Fig. 7:**
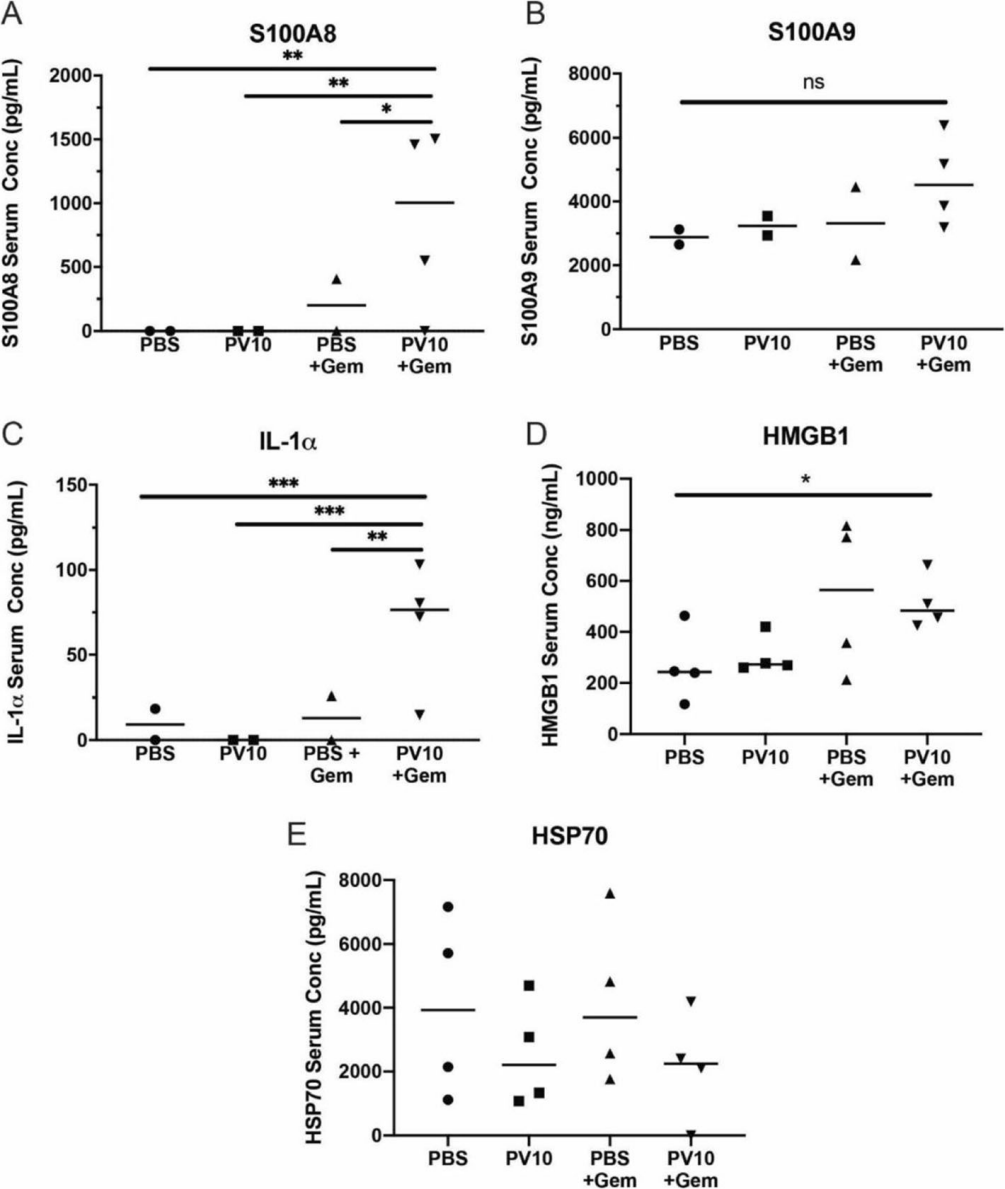
PV-10 and gemcitabine combination therapy increases the abundance of DAMPs in circulation. (**A**) S100A8, (**B**) S100A9, (**C**) IL-1α, (**D**) HMGB1, and (**E**) Hsp70 were measured in the sera of mice 9 days after the initiation of treatment. (*n* = 2–4 mice per group)

Next, we wanted to identify correlates that could explain the enhanced anti-tumor effect of gemcitabine when combined with PV-10. We analyzed the frequency of immune cells within the spleens of mice that received i.l. PV-10 and/or gemcitabine. We determined that the frequency of CD4 or CD8 T cells were unchanged by either PV-10 or gemcitabine (Fig. 6A). In contrast, gemcitabine effectively reduced the frequency of total CD11b^+^ myeloid cells in mice that received combination treatment with i.l. PBS or i.l. PV-10 (Fig. 6B). Specifically, gemcitabine reduced the frequency of Gr-1^+^ cells, while Gr-1^−^ cells comprised a higher proportion of myeloid cells in mice that received gemcitabine (Fig. 6C). Intralesional PV-10 treatment had no impact on the frequency of peripheral myeloid cells. Next, we examined the abundance of DAMPs in the serum of mice 9 days after receiving treatment and found that the tumors of mice treated with PV-10 and/or gemcitabine were significantly smaller than control tumors (Fig. 5). We identified that S100A8 and IL-1α were only elevated in mice that received both i.l. PV-10 and gemcitabine (Fig. 7A, C), while HMGB1 was elevated in mice receiving gemcitabine with or without PV-10 (Fig. 7D); S100A9 and Hsp70 was not significantly altered by PV-10 or gemcitabine (Fig. 7B, E). Thus, the anti-tumor activity of gemcitabine was associated with a persistent increase in HMGB1, and combinatorial i.l. PV-10 with systemic gemcitabine was associated with a persistent increase in S100A8, IL-1α, and HMGB1.

## Discussion

Combination therapy strategies with gemcitabine have often failed to improve the survival in patients with pancreatic cancer [15–18]. Notably, the addition of nab-paclitaxel has increased the survival of pancreatic cancer patients when treatment is combined with gemcitabine. However, the 2-year survival rate in patients that receive nab-paclitaxel plus gemcitabine is approximately 9% in comparison to 4% who received gemcitabine alone [18]. Thus, there is a potent need to define therapeutic combinations that enhance the durability of clinical responses that ultimately extend the survival of pancreatic cancer patients.

With the results described in this study, we believe that it is feasible to combine intralesional therapy with PV-10 with standard of care chemotherapy. We provide evidence that the potentiation of anti-tumor immune responses is necessary for tumor growth stabilization and regression. In Panc02 tumors that express an immunogenic neoantigen, OVA, we observed that PV-10 treatment alone or in combination with gemcitabine could induce the complete regression of treated OVA-expressing tumors and reduce the growth rate of distal uninjected tumors. However, the sensitivity of tumor cells to PV-10 monotherapy was lost in tumors that lack the expression of OVA. Indeed, gemcitabine monotherapy could reduce the size of Panc02 tumors that lack the expression of OVA, but this reduction in tumor growth was enhanced when gemcitabine was combined with intralesional PV-10 (Fig. 5C). We also observed that the increased tumor burden in mice with bilateral Panc02-OVA tumors responded differently to treatment. Specifically, PV-10 and gemcitabine were equally as effective at reducing the growth of a single Panc02-OVA tumor. However, the efficacy of gemcitabine, but not PV-10, was lost in mice bearing bilateral Panc02-OVA tumors. This suggests that the increased tumor burden in mice with lesions on both flanks diminished the efficacy of gemcitabine, which was overcome when combined with i.l. PV-10.

We next examined the efficacy of PV-10 combination therapy in Panc02 tumors that did not express OVA. We observed that PV-10 treatment alone had a modest effect in reducing tumor growth. Indeed, gemcitabine monotherapy effectively reduced tumor growth. However, the reduction of tumor growth was enhanced in mice that received combination therapy with PV-10 and gemcitabine (Fig. 5). Although Panc02 tumors are less immunogenic than Panc02OVA tumors, we were able to observe a significant improvement in tumor growth control and regression in mice that received combination therapy. Thus, the combination of gemcitabine with PV-10 can induce tumor regression even in less immunogenic tumors.

Treatment with gemcitabine is associated with the depletion of MDSCs and the promotion of tumoricidal activity by tumor-associated macrophages [24, 25]. Indeed, we observed that gemcitabine effectively reduced the frequency of bulk CD11b^+^ myeloid cells within spleens. However, there was a proportional shift characterized by the reduction of CD11b^+^Gr-1^+^ cells and an increase in CD11b^+^Gr-1^−^ myeloid cells. This reduction of cells was ultimately associated with reduced tumor growth in mice that received gemcitabine alone or the combination with PV-10. We further investigated systemic changes that could impact the immune system in response to PV-10 treatment. Indeed, PV-10 treatment alone increased the abundance of HMGB1 within 24 h after injection (Fig. 3B). We and others have shown that HMGB1 is an important mediator of DC activation and promotion of anti-tumor immunity [1, 6, 26]. Intriguingly, the increased abundance of HMGB1 and other DAMPs persisted in mice that received PV-10 in combination with gemcitabine (Fig. 7). Specifically, we observed that HMGB1, S100A8, and IL-1α were increased 9 days after treatment in mice that received PV-10 in combination with gemcitabine. Notably, mice that received the combination therapy exhibited the greatest reduction in tumor growth amongst all experimental groups, suggesting that the increased abundance of DAMPs in circulation is associated with better therapeutic responses. While HMGB1 can promote anti-tumor immune responses, it can simultaneously potentiate tumor cell survival mechanisms [27, 28]. Similarly, S100 proteins and IL-1α appear to have important roles in promoting pancreatic tumor progression. For instance, S100A8 and S100A9 enhance the production of IL-8 in pancreatic tumor cells, which could promote the accumulation of immunosuppressive myeloid cells, including MDSCs [29–31]. Meanwhile, IL-1α can enhance the metastatic potential of pancreatic tumor cells by maintaining the constitutive activation of nuclear factor κ-B (NFκB), promoting the secretion of hepatocyte growth factor (HGF), and can facilitate angiogenesis [32–34]. Future studies will address the individual contributions of these DAMPs on immunological responses and pro-tumorigenic mechanisms that take place during PV-10 treatment regimens.

We have previously shown that the combination of PV-10 can safely enhance the efficacy of anti-PD-1 immunotherapy in melanoma models [35]. In pancreatic cancer, immune checkpoint therapy is largely ineffective [36]. It is attractive to postulate that i.l. PV-10 could promote the efficacy of immune checkpoint therapy. However, it is possible that targeting known tumor-promoting mechanisms inherent to pancreatic cancer, such as long non-coding RNAs or the utilization of dasatinib could provide synergistic effects with PV-10 [37, 38].

While we have not measured specific parameters of toxicity in mice that received both PV-10 and gemcitabine, we did not observe any overt toxicity throughout the duration of our experiments. Treatment of cutaneous tumors in human subjects has been completed in multiple clinical trials with a manageable toxicity profile and previous studies have shown that PV-10 is tolerable in mice [39]. Indeed, the potential for drug interaction appears to be low since gemcitabine is excreted largely unmetabolized via the kidney [40], whereas PV-10 is excreted unmetabolized via the liver [41].

In conclusion, we demonstrate that intralesional therapy with PV-10 is a feasible strategy to augment therapeutic responses when combined with gemcitabine. Together, the results of this study provide support for future studies to investigate the induction of systemic anti-tumor immune responses after PV-10 treatment.

## Methods

### Cell lines and cell culture

Panc02 pancreatic cancer (obtained from ATCC), were cultured in RPMI media supplemented with 10% heat-inactivated FBS, 0.1 mM nonessential amino acids, 1 mM sodium pyruvate, 2 mM fresh L-glutamine, 100 mg/ml streptomycin, 100 U/ml penicillin, 50 mg/ml gentamicin, 0.5 mg/ml fungizone (all from Life Technologies, Rockville, MD), and 0.05 mM 2-ME (Sigma-Aldrich, St. Louis, MO). To generate the ovalbumin (OVA) expressing fluorescent Panc02 cell line, cells were exposed to supernatants containing a lentiviral vector comprised of a fluorescent ZsGreen (ZsG) protein and OVA. Upon successful transfection, ZsGreen^hi^ tumor cells were subjected to FACS using a BD FACSAria. OVA-ZsGreen^hi^ tumor cells were passaged in vitro 4 times whereby OVA expression was validated by staining for H2-K^b^ bound to SIINFEKL peptide (25-D1.16, BioLegend). CFPAC1, MiaPaca2, Panc-1, and SU8686 cells (obtained from ATCC) were grown and maintained in culture according to supplier guidelines. All cell lines tested negative for mycoplasma contamination and were passaged less than 10 times after initial revival from frozen stocks. All cell lines were authenticated using STR profiling in 2018.

### Apoptosis and cell death detection

Human and murine pancreatic tumor cells were cultured in 12 well plates and grown to ~ 60% confluency. Next, the indicated concentrations of PV-10 were added to media and cells were cultured for 24 h. Adherent cells were collected by gentle scraping and pooled with non-adherent cells. Cells were washed 3 times in PBS to remove excess PV-10. Washed cells were then stained with Annexin-V APC and DAPI (both from BioLegend) and analyzed on a BD FACSCelesta to determine the frequency of apoptotic and dead cells.

### Mouse models and treatment

Female C57BL/6 mice (6–8 weeks old) were purchased from Charles River Laboratories. Animal studies were carried out in compliance with ARRIVE guidelines. Mice were randomized before and after tumor implantation when put onto a study that included drug treatment. Panc02 and Panc02OVA-ZsGreen tumor cells (5 × 10^4^) were implanted subcutaneously into one flank of a mouse to establish a single tumor. To establish a bilateral tumor model, tumor cells were implanted in the opposite flanks. On day 7, a single tumor was treated with intralesional PV-10. Investigators could not be blinded to mice that received PV-10 due to the red staining of the tumor tissue and surrounding skin that was apparent within days after injection. Gemcitabine (60 mg/kg) was injected intraperitoneally twice per week for 2 weeks. Mice were housed at the Animal Research Facility of the H. Lee Moffitt Cancer Center and Research Institute. Animal studies were carried out in compliance with ARRIVE guidelines. Mice were observed daily and were humanely euthanized if a solitary subcutaneous tumor exceeded 300 mm^2^ in area, demonstrated evidence of ulceration, or if mice showed signs referable to metastatic cancer. Tumor growth measurement studies were concluded when > 30% of an experimental group required humane euthanization. Mice were humanely euthanized by CO_2_ inhalation followed by cervical dislocation according to the American Veterinary Medical Association Guidelines. All animal experiments were approved by the Institutional Animal Care and Use Committee and performed in accordance with the U.S. Public Health Service policy and National Research Council guidelines.

### Detection of DAMPs in mouse serum

Blood was collected at the termination of experiments. Blood specimens were centrifuged at 2000×g for 10 min at room temperature to separate serum from other blood content. The abundance of HMGB1 was determined by HMGB1 ELISA (IBL International); HSP70 and IL-1α were determined by Human/Mouse/Rat Total HSP70/HSPA1A DuoSet IC ELISA and Mouse IL-1 alpha/IL-1F1 Quantikine ELISA Kit (both from R&D Systems, a Biotechne brand); S100A8 and S100A9 were determined by Mouse Magnetic Luminex Assay (R&D Systems, a Biotechne brand) and analyzed on the Luminex 100 (LuminexCorp).

### Assessment of TLR4 activity by HEK-blue mTLR4 reporter cell line

Cryopreserved HEK-Blue mTLR4 cells (InvivoGen) were thawed, washed with pre-warmed medium (DMEM, 4.5 g/l glucose, 10% (v/v) FBS, 100 U/mL penicillin, 100 μg/mL streptomycin, 100 μg/mL Normocin, 2 mM L-Glutamine) and then transferred to a 25cm^2^ tissue culture flask containing 5 mL media. HEK-Blue mTLR4 cells were grown to 70–80% confluency and passaged twice before use in determining TLR4 activity. 2.5 × 10^4^ HEK-Blue mTLR4 cells were seeded in wells of a flat-bottom 96 well plate in medium containing 1X HEK-Blue Selection and 16% mouse serum taken from Panc02 tumor bearing mice treated with i.l. PBS or i.l. PV-10. Cultures were incubated for 18 h and TLR4 activity was determined by the detection of secreted embryonic alkaline phosphatase (SEAP) using a spectrophotometer at 655 nm.

### Flow cytometry

Tissues were prepared for flow cytometric analysis as previously described [42]. Briefly, spleens were harvested under sterile conditions and were homogenized by forcing the tissue through 100 μm cell strainers using the plunger from a syringe. Single-cell suspensions were prepared, and red blood cells were removed using red blood cell lysis buffer (BioLegend). The resulting suspension was passed through a 70 μm cell strainer and washed once with PBS. Cells were resuspended to a concentration of 0.5-1 × 10^6^ cells/mL for flow cytometric analysis in FACS Buffer containing PBS, 5% fetal bovine serum, 1 mM ethylenediaminetetraacetic acid (EDTA) (Sigma Aldrich), and 0.1% sodium azide (Sigma Aldrich). Cell viability was measured by staining cell suspensions with ZombieNIR (BioLegend). Prior to surface staining, cells were incubated with Fc Shield (TonboBiosciences) for murine specimens. For surface staining of murine specimens, cells were stained in FACS buffer with the following antibodies: CD3 (145-2C11), CD4 (GK1.5), CD8 (53–6.7), CD11b (M1/70), Gr-1 (RB6-8C5) (all from BioLegend). Fluorochromes that overlapped with the emission spectra of PV-10 were not used in this study. Cells were acquired by FACS Celesta (BD Biosciences), and the data were analyzed with FlowJo software (Tree Star).

### Statistical analysis

Graphs were generated using GraphPad Prism software. Graphs represent mean values with SEM. *P* values were calculated in each respective figure where statistical tests were indicated. For mouse-tumor growth studies, tumor growth curves are shown as mean with SEM and significance was determined by a 2-way ANOVA and Sidak’s multiple comparison’s test. Mice were randomized after tumor cell implantation into respective treatment groups. For all other experiments, data were compared using either an unpaired 2-tailed Student’s t-test corrected for multiple comparisons by a Bonferroni adjustment or Welch’s correction. * = *P* < 0.05; ** = *P* < 0.01; *** = *P* < 0.001; **** = *P* < 0.0001; ns = not significant.
